# Effects of Frenulotomy on Outcomes Associated with Breastfeeding Practice

**DOI:** 10.3390/jcm15020464

**Published:** 2026-01-07

**Authors:** Junsujee Wakhanrittee, Jiraporn Khorana, Siriphut Kiatipunsodsai

**Affiliations:** 1 Division of Pediatric Surgery, Department of Surgery, Faculty of Medicine, Thammasat University Hospital, Pathumthani 12120, Thailand; junsujee@tu.ac.th; 2 Division of Pediatric Surgery, Department of Surgery, Faculty of Medicine, Chiang Mai University Hospital, Chiang Mai 50200, Thailand; 3 Center of Clinical Epidemiology and Clinical Statistic, Faculty of Medicine, Chiang Mai University, Chiang Mai 50200, Thailand; 4 Clinical Surgical Research Center, Department of Surgery, Faculty of Medicine, Chiang Mai University, Chiang Mai 50200, Thailand; 5 Department of Biomedical Informatics and Clinical Epidemiology, Faculty of Medicine, Chiang Mai University, Chiang Mai 50200, Thailand

**Keywords:** tongue tie, ankyloglossia, frenulotomy, breastfeeding, LATCH score

## Abstract

**Background/Objectives**: This study aimed to evaluate the effects of frenulotomy in mother–infant pairs with problematic tongue-tie. **Methods**: A 2-year prospective observational cohort study was performed. Mother–infant pairs were divided into frenulotomy and non-frenulotomy groups by maternal choice. Four breastfeeding practice outcomes were evaluated: reduced latching pain scores, improved LATCH scores, regained birth weight within 2 weeks post-partum, and successful exclusive breastfeeding (EBF) at 3 months of age. The comparison between groups was performed using multivariable risk regression with propensity score analysis. **Results**: A total of 350 mother–infant pairs were included. There were 226 mother–infant pairs who underwent frenulotomy and 124 pairs in the non-frenulotomy group. The median latching pain scores significantly decreased from 6 to 3 at 24 h post-operatively and from 6 to 0 at 1 week post-operatively (*p* < 0.001). The median LATCH scores increased significantly from 5 to 9 at 1 week post-operatively (*p* < 0.001). LATCH scores within 2 weeks were improved in the frenolotomy group (risk ratio = 1.31, *p* = 0.017). The success rate of EBF at 3 months was 72.12% in the frenulotomy group and 76.61% in the non-frenulotomy group, with no statistically significance. **Conclusions**: Short-term breastfeeding outcomes and LATCH scores in mother–infant pairs with tongue-tie improved faster in those who underwent the procedure, with no complications.

## 1. Introduction

Tongue-tie, or ankyloglossia, is characterized by an unusually shortened, thickened, or tightened lingual frenulum that limits tongue motility. Some infants with tongue-tie cannot achieve good suckling, which results in breastfeeding difficulties. Several studies have demonstrated correlations between tongue-tie and breastfeeding problems, including maternal nipple pain, sore nipples, ineffective latch, poor infant weight gain, and inadequate milk supply [1,2,3,4]. These problems may cause some mothers to supplement breastfeeding with infant formula or to stop breastfeeding early.

Infant tongue-tie can be corrected by a frenulotomy, which can be performed under local anesthesia. This procedure is safe; complications attributed to it are rare and include bleeding requiring medical attention, scarring, infection, and salivary duct injury [5,6,7,8,9,10]. Several studies have reported significant improvement in breastfeeding problems following a frenulotomy, especially maternal nipple pain and latching scores [4,5,6,7,8,11,12,13,14]. O’shea and colleagues [9] reported a short-term reduction in nipple pain following a frenulotomy. Messner and colleagues [15] agreed with the potential benefit of a lingual frenulotomy on maternal nipple pain and with maternally reported improvement in breastfeeding. Few studies have shown its benefit on other outcomes such as infant weight or duration of breastfeeding.

Currently, there is insufficient evidence about how frenulotomy affects breastfeeding for mothers and infants. Outcomes such as infant weight, the rate and duration of breastfeeding, maternal nipple pain, and infant latch should be studied further. Therefore, this study aims to determine the positive impacts of frenulotomy on these aspects of breastfeeding, including reduced latching pain scores, improved LATCH scores, regained birth weight, and exclusive breastfeeding (EBF).

## 2. Materials and Methods

### 2.1. Patients

All mother–infant pairs who had evidence of tongue-tie were recruited. Inclusion criteria were (1) term newborns, (2) under the age of 72 h, and (3) tongue-tie confirmed by pediatric surgeons. Exclusion criteria were (1) infants and mothers who had contraindications for breastfeeding due to emergency or underlying conditions and (2) infants who had co-anomalies of the oral cavity, such as cleft lip or cleft palate.

### 2.2. Ethical Aspects

The study was approved by the Ethics Committee of Thammasat University (protocol code: MTU-EC-SU-1-213/61; date of approval: 9 October 2018). Recruited mothers were asked to sign a consent form on which the details of the study were clearly explained.

### 2.3. Study Outcomes

The present study measured four aspects of breastfeeding: reduced latching pain, improved LATCH scores, regained infant weight within two weeks after birth, and successful EBF at 3 months of age. The primary outcome was defined as an improved LATCH score, specifically one that had increased by 2 or more from the initial assessment by two weeks of age (a change considered clinically significant). The secondary outcomes were reduced latching pain, regained birth weight, and successful exclusive breastfeeding (EBF) at 3 months of age. Reduced latching pain was defined as a pain score that had decreased by 2 or more from the initial assessment by two weeks of age, which is also clinically significant. Regained birth weight was defined as infant weight increasing to the equivalent of birth weight within 14 days after birth. The proportion of mother–infant pairs in each study group who reached these target outcomes was collected.

### 2.4. Study Design and Definition

This prospective non-randomized cohort study was conducted from April 2019 to March 2021 in Thammasat University Hospital. In the first 24 h of life, all infants were assessed for the presence of tongue-tie by the postpartum nurse or pediatricians. If tongue-tie was identified, the mother- infant pairs were referred to the pediatric surgeons in the next 24–48 h to confirm the diagnosis and assess their breastfeeding problems. This period was marked as the initial assessment of the study. During the evaluation, baseline maternal and infant demographic data were recorded. Infant lingual frenula were evaluated and classified into 3 categories [2]. Mild tongue-tie was defined as a frenulum located in the proximal half of the tongue, moderate tongue-tie was defined as a frenulum located in the distal half of tongue but proximal to the fimbrinated fold, and severe tongue-tie was defined as a frenulum located at or distal to the fimbrinated fold.

Maternal nipples were examined, and the presence of sore nipples was recorded. Nipples were classified into 3 grades [16]. A normal nipple was defined as one with a length greater than or equal to 0.7 cm; a short nipple was defined as one with a length less than 0.7 cm; and flat or inverted nipple was defined as one that sank into the breast when the areola was pressed between thumb and forefinger. Mothers in whom the nipple on one side appeared different from the nipple on the other side were graded according to the more severe abnormality. All mothers were asked to commence breastfeeding for a short period to assess initial breastfeeding problems and the effectiveness of breastfeeding using the LATCH score [17]. Breastfeeding problems were recorded as latching pain, sore nipples, no latch-on (infant could not suck even though the mother was in a proper breastfeeding position), and improper latching (the infant’s tongue could not reach the areola and could not form an appropriate seal while suckling). LATCH scores range from 0–10; higher scores suggest greater breastfeeding efficacy. Latching pain was assessed using a scale ranging from 0–10; a score of 0 represents no pain, and a score of 10 represents the most severe pain [18].

In this study, the decision to recommend frenulotomy was reserved for mother–infant pairs who encountered obvious breastfeeding problems, including significant latching pain, severe nipple soreness, improper infant latching, or the inability to latch on even with proper positioning. The pediatric surgeon discussed the operation in detail with regard to the implications, benefits, and potential side effects, to facilitate a shared decision with the parents.

Following the initial assessment, the mother–infant pairs were non-randomly categorized into 2 groups based on treatment options: immediate frenulotomy or observation. Those who received immediate frenulotomy were re-assessed 24 h postoperatively for latching pain scores using the same tool as preoperatively. These mother–infant pairs were followed up 1 week postoperatively in the surgical clinic, where the surgical wound was examined. At this time, LATCH scores and latching pain scores were re-assessed.

Mother–infant pairs who were categorized for observation after the initial assessment were followed up 1 week later to reassess breastfeeding problems, LATCH scores, and latching pain scores. After the second assessment, frenulotomy was performed if indicated. Twenty-four hours postoperatively, latching pain was assessed by phone using the same tool. The same 1-week postoperative schedule was followed to assess surgical wounds, LATCH scores, and latching pain scores.

The infants who did not receive a frenulotomy after the second assessment were categorized as non-frenulotomy. Throughout the study period, both the frenulotomy and non-frenulotomy groups received similar care and support from the lactation consultants at our hospital.

### 2.5. Description of Procedure

The frenulotomy was performed by pediatric surgeons using a similar standard technique. 2% sterile lidocaine jelly was applied at the lingual frenulum. The tongue was elevated, and the exposed lingual frenulum was incised with a sterile Metzenbaum, severing the entire membranous part of the frenulum. After the procedure was finished, the infant was returned to the mother, and breastfeeding was immediately reinstated.

### 2.6. Follow-Up

At 2 weeks of age, the mother–infant pairs in both the frenulotomy and non-frenulotomy groups were scheduled for follow-up in the surgical clinic. The infants were weighed again, and the mothers were assessed for LATCH and latching pain scores. Finally, at the 3-month follow-up, each mother was contacted by phone and asked about the current pattern of the infant’s feeding and the duration of exclusive breastfeeding. Mothers who had stopped EBF before three months of age were asked for the reason for early cessation. EBF was defined as feeding an infant only breast milk and small amounts of ORS, vitamins, minerals, and medicines, but no non-human milk or food-based fluids [19].

### 2.7. Statistical Analysis

The sample size was calculated based on a 30-case pilot study of the mother–infant pairs in our hospital. The frenulotomy group had a 100% rate of regained infant weight, and the non-frenulotomy group had a regained infant weight rate of 93.33%. The test comparing two independent proportions was used with a significance level (α) of 0.05 and a power (β) of 0.80. The approximate sample size was at least 114 in each group.

The statistical analysis was performed Stata version 16.0 (StataCorp LLC, College Station, TX, USA). Categorical data were reported as counts and percentages and compared by Fisher’s exact test. Continuous data were evaluated for the normality using a histogram and the Shapiro–Wilk test. Normally distributed data were reported as mean ± standard deviation and compared by Student’s *t*-test. Non-normally distributed data were reported as median (interquartile range; IQR) and compared by the Wilcoxon rank-sum test. Ordinal data, including pain scores and LATCH scores, were reported as median (IQR). The comparison of pain scores and LATCH scores before and after frenulotomy was analyzed using the Wilcoxon signed-rank test. Comparisons between the frenulotomy and non-frenulotomy groups for the four outcomes associated with breastfeeding were analyzed using multivariable risk regression combined with a propensity score method known as inverse probability of treatment weighting (IPTW) and reported as a risk ratio (RR). For all comparisons, a *p* value < 0.05 was considered statistically significant.

The propensity score was calculated from the pre-treatment co-variate factors which might be the one of important considerations for the pediatric surgeons and/or the parents in making decision for a frenulotomy or some factors which influenced the chance for receiving this procedure. There were gender, gestational age, birth weight, tongue-tie grade, maternal age, nipple grade, parity, planned duration of breastfeeding, and initial breastfeeding problems, including sore nipples, neonatal jaundice, and excessive infant weight loss. Standardized mean differences of baseline covariates before and after IPTW and distribution of propensity scores for frenulotomy and non-frenulotomy groups are displayed in Supplementary File S1. The initial pain score and LATCH score were included in the final multivariable analysis process. These variables included baseline maternal-infant data, which are essential considerations for pediatric surgeons and parents when deciding on a frenulotomy.

## 3. Results

**Figure 1:**
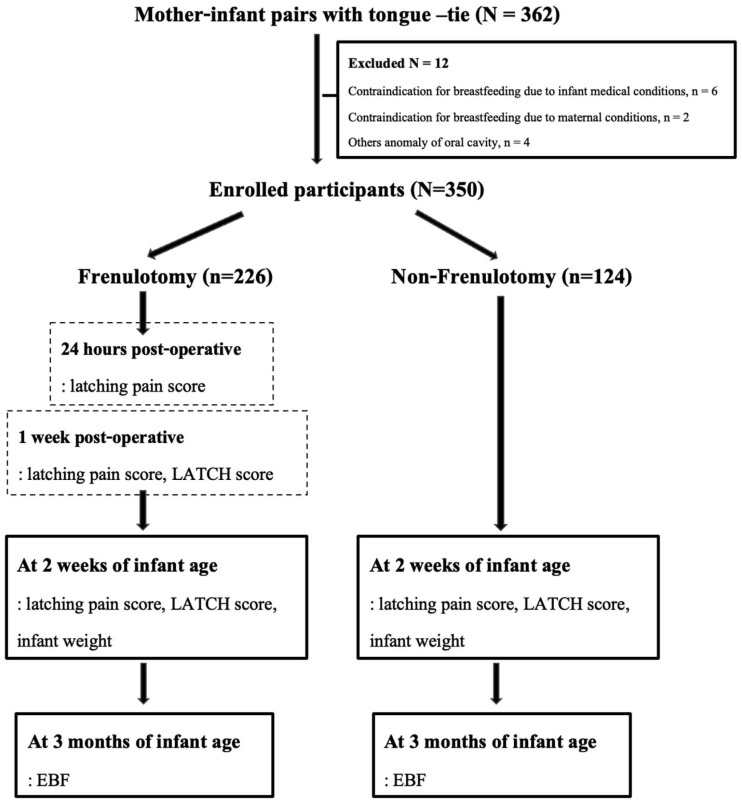
Study flow diagram.

Over the 2-year period, 350 mother–infant pairs who met the inclusion criteria were enrolled. There were 226 pairs in the frenulotomy group and 124 pairs in the non-frenulotomy group. The summarized study flow diagram is shown in Figure 1. Mother and infant baseline data are presented in Table 1. In the frenulotomy group, moderate to severe tongue-tie was recognized in most infants, with 53.10% of moderate degree and 46.02% of severe degree. The infants in the non-frenulotomy group had mild to moderate tongue-tie. Normal nipples were predominant in both groups, but the incidence of short and flat nipple types differed slightly.

Initial breastfeeding problems are summarized in Table 2. The mothers in the frenulotomy group reported significantly higher latching pain scores and lower LATCH scores. The majority of mothers (64.52%) in the non-surgical group had mild nipple pain, whereas 43.81% of those in the surgical group had moderate pain. Most mothers in our study (94.25%) reported an improper latch while breastfeeding, with a significantly higher proportion in the frenulotomy group.

During the procedure and in the 24 h afterward, there were no serious complications, including no significant bleeding and no Wharton’s duct injury. One-week post-operative surgical wounds were satisfactory in all infants, without any wound infection or scarring.

Univariable analysis of outcomes associated with breastfeeding practices is summarized in Table 3. At 2 weeks of infant age, both the individual results and the proportion of mother–infant pairs whose results reached the target change were compared. The medians of latching pain scores and LATCH scores were not different between the two groups. Proportions of pain reduction and LATCH score improvement were significantly greater in the frenulotomy group, with 73.45% of mothers reporting latching pain reduction and 95.13% reporting LATCH score improvement (*p* < 0.001). The means of infant weights and the proportion of infants whose body weight had regained were slightly greater in the frenulotomy group. At the 3-month follow-up, 73.71% of mother–infant pairs in this study achieved successful EBF. The success rate found in the frenulotomy group was lower than that in the non-surgical group, but this difference was not statistically significant. Several factors contributed to the cessation of EBF prior to 3 months of age. Insufficient breast milk supply was the leading cause (58.06%), followed by a combination of insufficient milk supply and personal factors (17.20%) and personal factors alone, such as the need to return to work (15.05%). Less frequent causes included very poor or absent latching (4.30%), severe breastfeeding pain (3.23%), and maternal illness (2.15%).

By multivariable risk regression using the IPTW method of propensity score analysis, mother–infant pairs in the frenulotomy group had an increased likelihood of LATCH score improvement within 2 weeks postpartum; the risk ratio was 1.31 (*p* =0.003), as shown in Table 4. Latching pain reduction was more likely in the frenulotomy group, with a risk ratio of 1.13, but this was not statistically significant. There was no significant effect of this procedure on regaining infant weight within 2 weeks postpartum or on the success rate of EBF at the 3-month follow-up.

For the frenulotomy group, the additional comparison of latching pain scores and LATCH scores between pre- and post-operatively is shown in Table 5. The median pain scores decreased significantly from 6 to 3 and 0 at 24 h and 1 week after frenulotomy, respectively (*p* < 0.001). The median LATCH scores also improved significantly from pre-procedure to 1 week post-procedure, increasing from 5 to 9 (*p* < 0.001).

## 4. Discussion

In the present study, latching pain scores decreased significantly after frenulotomy, indicating pain relief. LATCH scores also increased significantly after surgery, representing an improvement in breastfeeding efficacy for mothers. Both of these findings are similar to results reported by previous studies and support the benefits of frenulotomy when comparing pre- and post-procedure outcomes. Geddes et al. [4] reported that infants with tongue-tie tend to latch onto the nipple instead of latching onto the entire nipple-areolar complex and then compressing the nipple. After frenulotomy, nipple compression decreased, as confirmed by ultrasound, corresponding to improved latch and better milk transfer. Maternal complaints of latching pain indicate underlying poor latch, so optimizing the latch through frenulotomy can reduce nipple pain and may help improve breastfeeding efficacy. Frenulotomy can address up to three components of the LATCH score: latching on, audible swallowing, and comfort. When an infant achieves a better latch after surgery, the mother can hear regular, spontaneous sounds from the infant suckling and will feel comfortable and pain-free. These changes increase the overall LATCH score. The remaining two components, nipple grading and holding, cannot be corrected by surgery. However, good lactation support, which all mothers in this study received throughout the study period, may help alleviate some remaining problems.

The actual therapeutic effect of a frenulotomy should be assessed by comparison with a control group. Some bias may occur if the results are based solely on studies of a single population who underwent surgery. Therefore, the main objective of the present study was to assess the true treatment effect by comparing outcomes between the treatment group (mother–infant pairs undergoing frenulotomy) and the control group (those who did not). This study analyzed both short-term and medium-term outcomes to include all important aspects of breastfeeding. The short-term outcomes included a clinically important pain reduction rate, a LATCH score improvement rate, and the rate of regaining birth weight within 2 weeks postpartum. The EBF rate at 3 months of age served as the medium-term outcome. The authors consider that the change from the initial baseline assessment to the two-week follow-up for pain score and LATCH score is more important than the two-week follow-up score itself.

Breastfeeding efficacy is one of the interesting issues discussed in several studies. Although there were some differences in the measurement periods and tools used in each study, most studies show benefits of frenulotomy by comparing pre- and post-procedure outcomes, as did this study. Few studies have compared breastfeeding efficacy between surgery and non-surgery groups, and the overall results are still inconclusive. In a meta-analysis by O’Shea et al. [9] and a systemic review by Francis et al. [10], there was not enough evidence to conclude that frenulotomy improves efficacy. However, a study by Shekher et al. [20], which included several studies with different methodologies and measurement tools, reported a positive trend of frenulotomy on maternal breastfeeding. Buryk et al. [21] and Berry et al. [22] also demonstrated that mother–infant pairs who underwent frenulotomy were more likely to have better breastfeeding efficacy. In particular, a study by Berry, with outcomes quite similar to the present study, compared the proportions of mothers whose breastfeeding improved between surgery and non-surgery groups [22].

The LATCH score is an objective tool commonly used in both the healthcare system and our practice. It is simple and easy for postpartum nurses or general pediatricians to utilize for assessing maternal breastfeeding efficacy. Consistent with our findings, the LATCH scoring system has been employed as a study outcome in several previous studies [3,6,7,8,14], including work by Dollberg et al. [23] and Emond et al. [24]. While the methodology employed a non-randomized design, this prospective comparative study established internal validity through a large sample size (enhancing statistical power) and the use of propensity score analysis to control for potential confounding bias. The findings regarding breastfeeding efficacy are noteworthy: frenulotomy demonstrated a positive effect on LATCH score improvement, with the frenulotomy group showing a significantly greater likelihood of improvement within two weeks postpartum compared to the non-frenulotomy group. This outcome constitutes substantial evidence supporting the procedure’s advantage, suggesting that in cases where poor breastfeeding is primarily attributable to infant tongue tie, frenulotomy is a direct and effective treatment.

Similar to breastfeeding efficacy, there were a few clinical trials that compared latching pain between surgery and non-surgery groups [21,22,23,24]. Dollberg et al. [23] reported a greater decrease in pain scores after frenulotomy than after sham. Similarly, a study by Buryk et al. [21] demonstrated a significant reduction in nipple pain scores after intervention in both the frenulotomy and sham groups, but the improvement was greater in those who underwent procedure. The immediate improvement of pain following surgery suggests that the frenulotomy is a helpful method for relieving latching pain caused by tongue-tie, as demonstrated in the present study. However, this effect is not sustained in short-term comparisons. For instance, Emond et al. [24] reported no difference between randomized groups in median pain scores at 5-day and 8-week follow-ups. Similarly, this study found no difference in individual median pain scores between the surgery and non-surgery groups at two-weeks postpartum. Despite the additional analysis comparing the proportion of mothers who achieved a target pain reduction at two weeks, no statistically significant difference was found.

A retrospective study by Praborini et al. [25] demonstrated that frenulotomy improved latch and significantly improved infant weight, especially if surgery was performed before 8 days of age. Shah and colleagues [26] reported a positive correlation between post-partum LATCH scores greater than 6 and the likelihood of continued weight gain up to 6-weeks postpartum. The present study also compared the proportion of infants whose body weight returned by 14 days of age between the frenulotomy and non-frenulotomy groups. Latching improvement is likely to result in infants receiving adequate milk transfer with each suckling and mothers having enough breastmilk supply. Infant weight may be regained after correcting infant latch and improving maternal breastfeeding efficacy through surgery. However, the final analysis did not show a difference in this outcome between the two groups, although we found a positive effect of frenulotomy on LATCH score improvement. Several factors may affect infant weight, such as the types and amounts of milk received, which are not always linked to the quality of breastfeeding or latch. Additionally, individual maternal decisions regarding feeding patterns may have obscured the direct link between surgical correction and weight gain.

A medium-term outcome of the study was the breastfeeding rate at 3 months of infant age. The benefit of frenulotomy on this outcome is important and interesting, but it remains inconclusive. Few studies have directly compared breastfeeding rates between frenulotomy and non-frenulotomy groups. A study by Buryk demonstrated overall breastfeeding rates of 66% and 44% at 2 and 6 months of follow-up, but there was no difference between the surgery and sham groups in terms of the duration of breastfeeding [21]. Berry and colleagues reported an overall breastfeeding rate of 51% at 3 months of follow-up, but the researchers did not compare rates between the two randomized groups [22]. A study by Chi-Oi Lam and colleagues [27] showed a significantly higher direct breastfeeding rate in the frenulotomy group at 4 months of infant age; however, their analysis also showed a non-significant difference in the proportion of direct EBF. The present study demonstrated similar findings to previous studies, showing no statistically significant benefit of frenulotomy on the EBF rate.

Although a lower EBF success rate was observed in the frenulotomy group, we acknowledge that accurately estimating EBF rates and duration requires medium- to long-term follow-up, which makes controlling for confounding factors especially difficult. The main reasons for discontinuing EBF before 3 months of age, as described in the literature, were insufficient milk supply, personal factors (such as the need to return to work), and a combination of the two. It is notable that discontinuation due to personal factors was more frequent in the frenulotomy group, accounting for 66.67% of mothers who cited these reasons. Considering the socio-cultural environment of Thailand, where such personal reasons for EBF cessation are often unavoidable or difficult to avoid, this factor may partially explain the lower proportion of EBF documented in the frenulotomy group. However, the observed difference was not statistically significant.

In our setting, clinical decisions regarding frenulotomy are recommended for mother–infant pairs who encounter obvious breastfeeding problems caused by tongue-tie, but the final therapeutic choice requires a shared discussion between the pediatric surgeon and the parents. Several factors should be considered, including the anatomical grade of tongue-tie, maternal nipple anatomy, individual breastfeeding efficacy, severity of detected breastfeeding problems, and the adequacy of lactation support. Our findings indicate that only three infants with severe tongue tie remained in the non-surgical group, whereas two infants with mild tongue tie underwent surgery. Further details regarding these cases are also provided in the Supplementary File S2. Although a prospective observational design carries a greater susceptibility to bias and confounding by indication compared to a randomized controlled study, this approach was employed to maintain ethical integrity and acknowledge the unique cultural constraints inherent to the Thai healthcare system. Randomization in this context is difficult and challenging, particularly considering Thai parenthood culture. Furthermore, adopting a design consistent with actual clinical practice ensures that the findings possess enhanced external validity and real-world applicability.

To minimize the inherent limitations of the prospective cohort design, the authors applied the propensity score analysis to balance pre-treatment confounders. However, some residual bias persists, likely due to unmeasured factors that were not identified before treatment. Moreover, many uncontrolled factors may affect the results, particularly those requiring a longer follow-up period. To definitively determine the true efficacy of frenulotomy across all breastfeeding outcomes, a randomized controlled trial (RCT) is the best design. Future randomized research should consider focusing on a single outcome or on the severity of the tongue-tie.

Another limitation of this study is the inconsistency between subjective outcomes, such as LATCH and pain scores, and objective indicators, such as weight gain and exclusive breastfeeding (EBF) rates. While the LATCH scoring system helps assess the breastfeeding process, its subjective nature makes it susceptible to observer bias and situational volatility, thus potentially lacking the strong predictive power of objective indicators. This underscores the risk of relying solely on observational scoring systems; despite their widespread use, caution should be exercised when using them to predict long-term clinical success without validating data.

## 5. Conclusions

Frenulotomy benefits mother–infant pairs who have breastfeeding problems associated with infant tongue-tie. Maternal latching pain was significantly reduced within 24 h and continued to improve at 1 week after surgery. A positive effect of frenulotomy on short-term breastfeeding outcomes, such as LATCH score improvement within 2 weeks postpartum, was also demonstrated. The benefits in terms of regained birth weight and the exclusive breastfeeding (EBF) rate were not significantly different between the surgical and non-surgical groups. More randomized trials should be conducted.

## Figures and Tables

**Table 1:** Baseline data of mothers and infants between the frenulotomy group and non-frenulotomy group.

Baseline Data	Frenulotomy (n = 226)	Non-Frenulotomy (n = 124)	*p*-Value	STD
1. Infant data				
Gender				
Female	77 (34.07%)	61 (49.19%)	0.006	−0.309
Male	149 (65.93%)	63 (50.81%)		
Gestation Age (week)	38.37 ± 0.94 ^a^	38.32 ± 0.98 ^a^	0.646	−0.051
Birth weight (gram)	3154.28 ± 353.61 ^a^	3124.77 ± 395.26 ^a^	0.474	−0.079
≥2500	221 (97.79%)	120 (96.77%)	0.726	0.062
<2500	5 (2.21%)	4 (3.23%)		
Tongue tie grade				
mild	2 (0.88%)	64 (51.61%)	<0.001	−1.771
moderate	120 (53.10%)	57 (45.97%)		
severe	104 (46.02%)	3 (2.42%)		
2. Maternal data				
Maternal age (year)	30.32 ± 5.73 ^a^	29.55 ± 5.05 ^a^	0.216	−0.141
<20	4 (1.77%)	0	0.147	−0.095
20 to <35	165 (73.01%)	100 (80.65%)		
≥35	57 (25.22%)	24 (19.35%)		
Nipple grade				
Normal	170 (75.22%)	102 (82.26%)	0.011	−0.080
Short	54 (23.89%)	17 (13.71%)		
Flat/invert	2 (0.88%)	5 (4.03%)		
Parity				
Primiparity	124 (54.87%)	63 (50.81%)	0.502	−0.081
Multiparity	102 (45.13%)	61 (49.16%)		
Planned duration of breastfeeding				
-0–3 months	20 (8.85%)	13 (10.48%)	0.320	−0.135
-3–6 months	33 (14.60%)	25 (20.16%)		
->6 months	173 (76.55%)	86 (69.35%)		

^a^: mean ± standard deviation, STD; Standardized mean difference.

**Table 2:** Initial breastfeeding problems.

Breastfeeding Problems	Frenulotomy (n = 226)	Non-Frenulotomy (n = 124)	*p*-Value	STD
Cannot latching	24 (10.62%)	8 (6.45%)	0.246	−0.149
Inadequate/Improper latching	213 (94.25%)	86 (69.35%)	<0.001	−0.679
Latching pain score	5 (3–6) ^b^	3 (0–5) ^b^	<0.001	−0.742
no or mild (0–3)	71 (31.42%)	80 (64.52%)	<0.001	−0.697
moderate (4–6)	99 (43.81%)	33 (26.61%)		
severe (7–10)	56 (24.78%)	11(8.87%)		
LATCH score	6 (5–6) ^b^	7 (3–9) ^b^	<0.001	0.866
less than 8	216 (95.58%)	83 (66.94%)	<0.001	0.786
equal or more than 8	10 (4.42%)	41 (33.06%)		
Sore nipple	41 (18.14%)	15 (12.10%)	0.170	−0.169
Excessive infant weight loss at 48 h of age	44 (19.47%)	24 (19.35%)	1.000	−0.003
Neonatal jaundice at 48 h of age	32 (14.16%)	14 (11.29%)	0.510	−0.086

^b^: median (interquartile range), *p*-value from Wilcoxon sign-rank test, STD; Standardized mean difference.

**Table 3:** Univariable analysis of outcomes associated with breastfeeding practice.

Outcomes	Frenulotomy Group (n = 226)	Non-Frenulotomy Group (n = 124)	*p*-Value
At 2 weeks of infant age			
Infant weight (gram)	3568.40 ± 388.26	3482.91 ± 395.17	0.051
Regained birth weight (n,%)	219 (96.90%)	117 (94.35%)	0.263
Latching pain score	0 (0–2) ^b^	0 (0–2) ^b^	0.674
Latching pain reduction (n,%)	116 (73.45%)	54 (43.55%)	<0.001
LATCH score	9 (8–10) ^b^	9.5 (8–10) ^b^	0.416
LATCH score improvement (n,%)	215 (95.13%)	86 (69.35%)	<0.001
At 3 months of infant age			
EBF (n,%)	163 (72.12%)	95 (76.61%)	0.377

^b^: median (interquartile range), *p*-value from Wilcoxon sign-rank test, EBF; Exclusive Breastfeeding.

**Table 4:** Multivariable risk regression with the Inverse probability of treatment weighting (IPTW) analysis for the effect of the frenulotomy.

Outcomes	Adjusted Risk Ratio (RR)	95% Confidence Interval	*p*-Value
At 2 weeks of infant age			
Regained birth weight	1.09	0.98–1.20	0.101
Latching pain reduction	1.13	0.76–1.69	0.523
LATCH score improvement	1.31	1.09–1.59	0.003
At 3 months of infant age			
EBF	1.06	0.87–1.29	0.544

EBF; Exclusive Breastfeeding.

**Table 5:** The comparison of latching pain score and LATCH score between pre- and post-frenulotomy periods.

Outcome(s)	Pre-Frenulotomy	24 h Post-Frenulotomy	*p*-Value	1 Week Post-Frenulotomy	*p*-Value
Latching pain score ^b^	6 (5–8)	3 (2–5)	<0.001	0 (0–3)	<0.001
LATCH score ^b^	5 (5–6)	NA	NA	9 (8–10)	<0.001

^b^: median (interquartile range), *p*-value from Wilcoxon sign-rank test, NA; Not Applicable.

## Data Availability

The data presented in this study are available on request from the corresponding author.

## Supplementary Materials

The following supporting information can be downloaded at: https://www.mdpi.com/article/10.3390/jcm15020464/s1, Supplementary File S1: Standardized Mean Differences of Baseline Covariates Before and After IPTW, Supplementary S2: The classification of infant tongue-tie by treatment groups and cases description.

## Abbreviations

The following abbreviations are used in this manuscript:

EBF	Exclusive Breastfeeding
IPTW	Inverse probability of treatment weighting
ORS	Oral rehydration salts
STD	Standardized difference
