# Supplementary material for: Effects of Frenulotomy on Outcomes Associated with Breastfeeding Practice
Source: J Clin Med. 2026 Jan 7;15(2):464. doi: 10.3390/jcm15020464 (PMC12841757; doi:10.3390/jcm15020464)

Standardized Mean Differences of Baseline Covariates Before and After IPTW

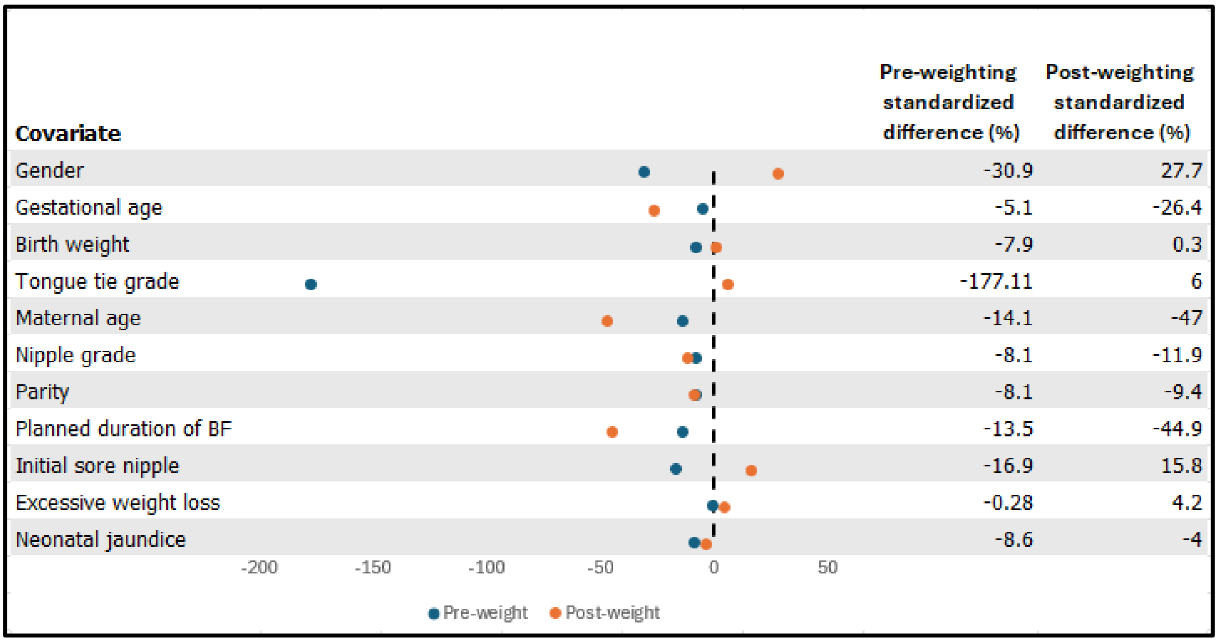

Distribution of Propensity Scores for Frenulotomy and Non-Frenulotomy Groups

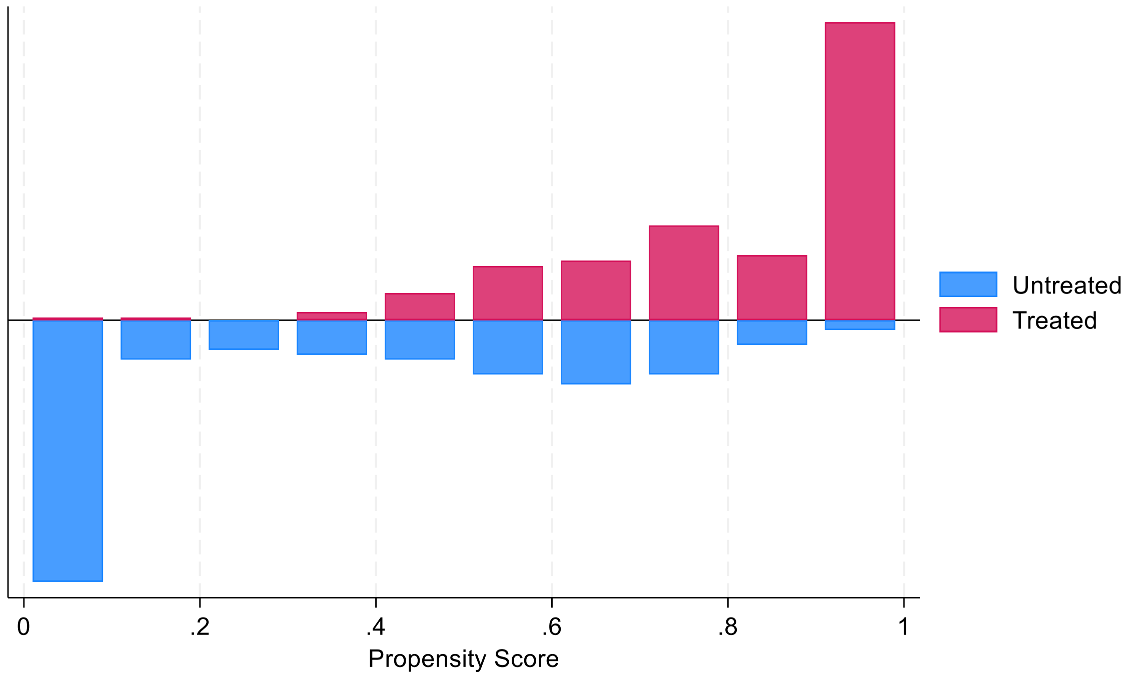

Supplement: Supplementary file 1 [file jcm-15-00464-s001.zip › Supplementary file/Supplementary file 1.pdf]
