# Supplementary material for: Effects of Frenulotomy on Outcomes Associated with Breastfeeding Practice
Source: J Clin Med. 2026 Jan 7;15(2):464. doi: 10.3390/jcm15020464 (PMC12841757; doi:10.3390/jcm15020464)
Supplement: Supplementary file 1 [file jcm-15-00464-s001.zip › Supplementary file/Supplementary file 2.pdf]

Supplementary file no.2

Table: The classification of infant tongue-tie by treatment groups.

Tongue-Tie grade	Non-frenulotomy group (n,%)	Frenulotomy group (n,%)
- Mild	64 (96.67)	2 (3.03)
- Moderate	57 (32.20)	120 (67.80)
- Severe	3 (2.80)	104 (97.20)

1. The following case series describes three infants diagnosed with severe tongue-tie who did not undergo frenulotomy.

Case No. 1: During the initial assessment, the mother reported a complete inability to latch, which was reflected in a LATCH score of 6. Due to poor latching and insufficient milk supply, the mother decided to switch to exclusive formula feeding at three days after birth and declined a frenulotomy. At the 2-week postpartum follow-up, latching difficulties and LATCH scores remained unimproved. The infant was continuously fed with formula and regained body weight at a normal rate. However, exclusive breastfeeding (EBF) was not achieved by the 3-month follow-up.

Case No. 2: At the initial assessment, the mother reported a nipple pain score of 3 and a LATCH score of 6. By the 2-week postpartum follow-up, the pain score was 4, while the LATCH score had improved to 8. Because breastfeeding issues showed progress over time, a frenulotomy was not recommended. The infant remained exclusively breastfed from birth through the 3-month follow-up, with normal weight gain achieved. Notably, the mother's prior breastfeeding experience likely provided the necessary skills and support to optimize breastfeeding efficacy with her current infant.

Case No. 3: The mother had extensive breastfeeding experience from three previous children. Initial assessment revealed a nipple pain score of 3 and a LATCH score of 8, indicating good breastfeeding efficacy. Consequently, a frenulotomy was not indicated. By 2 weeks postpartum, the LATCH score had increased to 10, and the mother reported being pain-free during feeding. The infant successfully achieved exclusive breastfeeding (EBF) by the 3-month follow-up.

2. The following cases involve two infants with mild tongue-tie who underwent a frenulotomy.

Case No. 1: At the initial assessment, the mother reported a nipple pain score of 2 and a LATCH score of 7; a frenulotomy was not indicated at that time. However, the procedure was performed during the second assessment as symptoms persisted at a similar intensity and the parents requested intervention. By 2 weeks postpartum, the LATCH score had increased to 9, and exclusive breastfeeding (EBF) was successfully achieved. This success was maintained through the 3-month follow-up.

Case No. 2: At the initial assessment, the mother reported a nipple pain score of 4 and a LATCH score of 5, indicating ineffective breastfeeding. Although intervention was suggested, the parents declined a frenulotomy at that time. At the subsequent follow-up, breastfeeding difficulties persisted without improvement; consequently, a frenulotomy was performed. By 2 weeks postpartum, the LATCH score had improved to 8, and the mother

reported being pain-free during latching. Exclusive breastfeeding (EBF) was successfully achieved and maintained through the three-month follow-up.
